# Study on the correlation between gene polymorphisms of adiponectin and resistin levels and abdominal aortic aneurysm

**DOI:** 10.1016/j.clinsp.2023.100298

**Published:** 2023-10-26

**Authors:** Zhongyin Wu, Shuangqing Liu, Zhi Yang, Xiangxi Meng, Yang An, Hong Zhang

**Affiliations:** a Department of Vascular Surgery, Affiliated Hospital of Chengde Medical University, Chengde, Hebei, China; b Department of Emergency, Affiliated Hospital of Chengde Medical University, Chengde, Hebei, China; c Department of Burn Plastic Surgery, Affiliated Hospital of Chengde Medical University, Chengde, Hebei, China

**Keywords:** Gene polymorphisms, Adiponectin, Resistin, Abdominal aortic aneurysm, Genetic susceptibility

## Abstract

**Objectives:**

Abdominal Aortic Aneurysm (AAA) is a complex disease with both genetic and environmental risk factors. This study aimed to examine the potential association of the +276G/T and -420C>G polymorphisms in the resistin gene with AAA susceptibility and progression.

**Method:**

We performed a retrospective study involving AAA patients and healthy controls, assessing the distribution of the +276G/T and -420C>G genotypes in both groups. Hardy-Weinberg equilibrium was assessed for both polymorphisms. Logistic regression was used to explore the influence of these genotypes on AAA occurrence and progression, adjusting for relevant confounders.

**Results:**

The distribution of +276G/T polymorphism did not significantly differ between AAA patients and controls. Conversely, a significant difference was observed in the genotype distribution of -420C>G polymorphism between the two groups. The CC genotype and CC/CG genotypes of -420C>G polymorphism were found to be associated with an increased risk and progression of AAA.

**Conclusions:**

The -420C>G polymorphism, particularly the CC genotype and CC/CG genotypes, might play a substantial role in AAA susceptibility and progression. The present findings underscore the need for further investigations to confirm these associations and fully elucidate the role of the resistin gene in AAA.

## Highlights

• Examined resistin gene polymorphisms' link with AAA.
• -420C>G (especially CC/CG) tied to AAA risk & progression.
• +276G/T polymorphism showed no AAA association.
• Resistin gene's potential role in AAA highlighted.

## Introduction

Abdominal Aortic Aneurysm (AAA) is a potentially lethal vascular disease characterized by the progressive and permanent dilation of the abdominal aorta.^1^ It remains an area of significant public health concern, due to its typically asymptomatic nature until rupture, which is associated with high mortality rates.^2^ While advancements have been made in the diagnosis, monitoring, and treatment of AAA, the lack of early symptoms makes the disease often detected at advanced stages. Therefore, understanding the various biological and genetic markers that could contribute to the early detection and therapeutic strategies of AAA is vital.

Adipokines, primarily secreted by adipose tissue, have been implicated in a wide range of physiological processes including inflammation, atherosclerosis, and regulation of metabolic homeostasis, all of which have been associated with cardiovascular diseases.^3^, ^4^, ^5^ Among these, adiponectin and resistin stand as interesting molecules to investigate in the context of AAA due to their distinct roles in cardiovascular health. Adiponectin is an adipocyte-derived protein that exerts anti-inflammatory and anti-atherosclerotic effects, promoting endothelial function, and inhibiting vascular smooth muscle cell proliferation.^6^^,^^7^ However, contradicting its protective properties, lower plasma levels of adiponectin have been associated with increased cardiovascular risk in numerous studies.^8^^,^^9^ Resistin, a hormone primarily secreted by adipocytes, exhibits contrasting properties to adiponectin. It has been associated with pro-inflammatory and atherosclerotic activities, implying its role as a potential risk factor in cardiovascular diseases.^10^^,^^11^ Moreover, genetic polymorphisms in the genes encoding adiponectin and resistin could modify their production, secretion, or biological activity. These genetic alterations could potentially contribute to AAA's pathogenesis, providing a novel approach to understanding the disease mechanism.

In light of these insights, this paper aims to investigate the correlation between adiponectin and resistin levels, their gene polymorphisms, and AAA. We hope to shed light on the potential roles of these adipokines and their genetic variations in the pathophysiology of AAA. The outcomes could guide the development of new therapeutic strategies and early detection methods for this devastating disease.

## Materials and methods

### Patient data

In the design, execution, and reporting of this observational study, we adhered to the guidelines set forth by the STROBE (Strengthening the Reporting of Observational Studies in Epidemiology) Statement. This study was conducted with the approval of the institutional review board of the Affiliated Hospital of Chengde Medical University and in accordance with the Helsinki Declaration (protocol number: CYFYLL2019402). All patients signed informed consent. The data of 200 patients who underwent elective surgical repair for AAA from October 2016 to December 2021 was reviewed. The inclusion criteria were as follows: 1) Patients aged 50 years or older; 2) Patients diagnosed with AAA based on the criteria of an aortic diameter ≥3.0 cm or an aortic diameter ≥1.5 times the normal diameter; 3) Patients willing to provide blood samples for genetic and biochemical analyses. Exclusion criteria included: 1) Presence of other cardiovascular diseases such as coronary artery disease, peripheral artery disease, and cerebrovascular disease; 2) Presence of acute or chronic infectious diseases, autoimmune diseases, or malignancies; 3) Patients who have undergone previous abdominal aortic surgery; 4) Patients taking medications known to affect adiponectin or resistin levels (such as thiazolidinediones and statins) within three months prior to the study; 5) Patients with end-stage renal disease or liver disease; 6) Patients who declined to participate in the study.

A control group of 200 age and sex-matched individuals without AAA, but with similar comorbidities, were also reviewed from outpatient clinics during the same period. The inclusion and exclusion criteria for the control group were the same as for the AAA group, excluding the criterion related to the presence of AAA. Data collected from each participant included demographic characteristics (age, sex), anthropometric measurements (weight, height, body mass index), smoking and drinking habits, medical history, and medications. Blood samples were also collected from all participants for biochemical and genetic analyses.

## Research methods

### Blood sample collection

Peripheral blood samples were collected from all participants after an overnight fast. The samples were immediately centrifuged, and the plasma was separated and stored at -80°C until analysis. DNA was also extracted from peripheral blood leukocytes using standard protocols for genetic analysis.

### Genetic analysis

(1) Genomic DNA Extraction: Genomic DNA was extracted from the peripheral blood leukocytes of all participants using a PureLink™ Genomic DNA Mini Kit (Thermo Fisher Scientific, USA) as per the manufacturer's instructions. The quality and quantity of the extracted DNA were assessed using a NanoDrop™ spectrophotometer (Thermo Fisher Scientific, USA). High-quality DNA samples with an A260/280 ratio of 1.8–2.0 were considered suitable for further analysis and were stored at -20°C until use. (2) Selection of Single Nucleotide Polymorphisms (SNPs): Based on a comprehensive review of the literature, we selected two SNPs for this study due to their reported associations with cardiovascular diseases and potential functional significance. These included the SNP at position +276 in the adiponectin gene (referred to as +276G/T), and the SNP at position -420 in the resistin gene (referred to as RETN -420C>G). (3) Polymerase Chain Reaction (PCR) Amplification and Sequencing: The regions of interest in the adiponectin and resistin genes containing these SNPs were amplified by PCR using specific primers. The PCR conditions were optimized to ensure specific amplification. PCR products were then purified using a QIAquick PCR Purification Kit (QIAGEN, Germany). Sequencing of the purified PCR products was carried out using the ABI PRISM® 3730 DNA Analyzer (Applied Biosystems, USA). The obtained sequences were then compared with the reference sequences from the NCBI GenBank database to identify the SNPs. (4) Genotype Determination: Genotype determination for each SNP was carried out by two independent investigators blinded to the clinical data. Any discrepancies were resolved by consensus, with a re-sequencing of the sample conducted if necessary.

### Diameter of blood vessel

The diameter of the blood vessel was professionally measured by a sonographer. The measurement standard applied was the intima-to-intima diameter of the subrenal abdominal aorta, specifically 1 cm below the renal arteries. According to Akai's classification criteria for AAA: AAA < 50 mm is a small aneurysm; AAA ≥45 mm is a growing aneurysm.

### Statistical analysis

Baseline characteristics of the participants were presented as mean ± standard deviation for continuous variables and frequencies (percentages) for categorical variables. Differences between the AAA and control groups were analyzed using the Student's t-test for continuous variables and the Chi-Square test or Fisher's exact test for categorical variables, as appropriate. The association between adiponectin and resistin levels, their gene polymorphisms, and AAA was assessed using multivariable logistic regression analysis. Adjustments were made for potential confounding variables such as age, sex, Body Mass Index (BMI), smoking status, and comorbidities. A p-value of less than 0.05 was considered statistically significant. All statistical analyses were performed using SPSS 22.0 (IBM Corp., Armonk, NY, USA).

## Results

### Baseline characteristics of two groups

The AAA patient group and the control group were comparable in terms of age and sex, ensuring that the present study population was well-matched. The mean age was 68.5 years in the AAA group and 67.8 years in the control group. The sex distribution was also similar, with a slightly higher proportion of males in both groups. There was a slightly higher BMI on average in the AAA group compared to the control group. Smoking habits were more prevalent in the AAA group, with two-thirds of the patients having a history of smoking, compared to approximately half in the control group. In terms of medical history, hypertension was slightly more common in the AAA group, with over 70% of patients suffering from this condition. Diabetes and dyslipidemia were also present in a proportion of both groups, with a slightly higher incidence in the AAA group (Table 1).

**Table 1:** Baseline characteristics of two groups.

Characteristics	AAA Patients (n = 200)	Controls (n = 200)
Age (years)	68.5 ± 8.7	67.8 ± 8.1
Sex (M/F)	190/110	195/105
BMI (kg/m^2^)	28.3 ± 3.9	27.5 ± 4.2
Smoking (Yes/No)	200/100	185/115
Alcohol use (Yes/No)	120/180	110/190
Hypertension (Yes/No)	215/85	200/100
Diabetes (Yes/No)	90/210	80/220
Dyslipidemia (Yes/No)	160/140	150/150

Note: The results are presented as mean ± standard deviation for continuous variables and number for categorical variables.

### Hardy-Weinberg analysis of two groups

The Hardy-Weinberg equilibrium was assessed for the genotype distributions of two polymorphisms, +276G/T and -420C>G, in both the study and control groups. The chi-square test was applied to compare observed and expected genotype distributions under the Hardy-Weinberg principle. For the +276G/T polymorphism, the Chi-Square value was 0.196, with a corresponding p-value of 0.908. For the -420C>G polymorphism, the chi-square value was 0.367, and the p-value was 0.832. In both cases, the p-values exceeded the significance threshold of 0.05, indicating no significant deviation of observed genotype distributions from Hardy-Weinberg equilibrium. This suggests that the present samples are likely representative of a larger Mendelian population, affirming the validity and representativeness of the present study population for these loci (Table 2).

**Table 2:** Hardy-Weinberg analysis of two groups.

Item	+276G/T			-420C>G		
	GG	TT	GT	CC	CG	GG
Observed Numbers	211	153	36	166	169	65
Expected Numbers	205	157	38	171	170	59
χ²		0.192			0.367	
P		0.908			0.832	

### Genotype distributions of two groups

The genotype distributions of the +276G/T and -420C>G polymorphisms were compared between the AAA patients and the control group. For the +276G/T polymorphism, the Chi-Square test revealed no significant difference in the genotype distributions between the AAA patients and the control group (χ² = 0.552, p = 0.759).In contrast, for the -420C>G polymorphism, a significant difference in the genotype distributions was observed between the two groups (χ² = 7.035, p = 0.030). These results indicate that while the genotype distribution of +276G/T polymorphism does not differ significantly between the AAA patients and the control group, the distribution of the -420C>G polymorphism does, suggesting a potential role of this polymorphism in AAA (Table 3).

**Table 3:** Genotype distributions of two groups.

Groups	+276G/T			-420C>G		
	GG	TT	GT	CC	CG	GG
AAA Patients	102	80	18	89	71	40
Controls	109	73	18	79	95	26
χ²		0.552			7.035	
P		0.759			0.030	

### Influence of genotype of -420C>G on the occurrence of AAA

The influence of the -420C>G genotype on the occurrence of AAA was assessed using logistic regression, with the CG genotype as the reference category. Compared with the CG genotype, the CC genotype was significantly associated with an increased risk of AAA (p = 0.014). However, the GG genotype did not show a significant association with AAA risk (p = 0.304). When comparing the combined CC/CG genotype against the reference CG genotype, there was a significant increase in AAA risk (p < 0.001). In contrast, the combined CC/GG genotype did not show a significant association with AAA risk when compared to the reference CG genotype (p = 0.090). These results indicate that the -420C>G polymorphism in the resistin gene might play a significant role in the occurrence of AAA, particularly for the CC genotype and the combined CC/CG genotypes (Table 4).

**Table 4:** Influence of genotype of -420C>G on the occurrence of AAA.

Genotype	β	SE	OR*	95% CI		p
G	1.000 (ref)	‒	‒	‒	‒	‒
CC	0.380	0.155	1.463	1.079	1.984	0.014
GG	0.146	0.142	1.157	0.876	1.528	0.304
CC/CG	0.957	0.156	2.605	1.917	3.540	0.000
CC/GG	0.686	0.405	1.985	0.898	4.388	0.090

Note: The results are adjusted by correct age, gender, smoking, drinking, hypertension, diabetes, dyslipidemia.

### Influence of genotype of -420C>G on the progression of AAA

Table 5 presents the influence of the -420C>G genotype on the progression of AAA. Using the CG genotype as the reference category, the logistic regression analysis revealed that the CC genotype had an Odds Ratio (OR) of 1.767 (p = 0.225), and the GG genotype had an OR of 1.896 (95% CI, 0.940 to 3.824; p = 0.074). Notably, the CC/CG genotype exhibited a significant impact on AAA progression with an OR of 3.235 (p = 0.030). On the other hand, the CC/GG genotype showed a tendency toward influencing AAA progression with an OR of 2.975, but this was not statistically significant (p = 0.150). Therefore, the data suggest that certain -420C>G genotypes may have a role in the progression of AAA, with the CC/CG genotype showing a significant association.

**Table 5:** Influence of genotype of -420C>G on the progression of AAA.

Genotype	β	SE	OR*	95%CI		p
CG	1.000 (ref)	‒	‒	‒	‒	‒
CC	0.569	0.470	1.767	0.704	4.435	0.225
GG	0.640	0.358	1.896	0.940	3.824	0.074
CC/CG	1.174	0.539	3.235	1.124	9.311	0.030
CC/GG	1.090	0.758	2.975	0.674	13.131	0.150

Note: The results are adjusted by correct age, gender, smoking, drinking, hypertension, diabetes, dyslipidemia.

## Discussion

Abdominal Aortic Aneurysm, a complex and multifactorial disease, holds a cryptic genetic landscape that researchers have long sought to unravel. While multiple genetic variants have been associated with AAA, their individual contributions and interactions remain largely enigmatic. However, one notable limitation of prior research is the predominant focus on individual gene expression levels, often overlooking potential gene-gene and gene-environment interactions that could be central to AAA pathogenesis.^12^ AAA's genetic underpinnings extend beyond a simple monogenic model, encompassing a spectrum of numerous genes each contributing modestly to the overall risk.

Past research has highlighted the relevance of genetic variants in inflammatory and extracellular matrix genes in AAA's pathogenesis. Variants in the MMP-3 gene, for instance, which encodes a matrix metalloproteinase involved in extracellular matrix remodeling, have been associated with AAA risk.^13^ Similarly, polymorphisms in the IL-6 gene, a key regulator of inflammatory responses, have been implicated in AAA.^14^ Nevertheless, some studies have suffered from small sample sizes, limiting their power to detect subtle associations and potential confounding factors.^15^ Furthermore, a majority of previous studies have been conducted in homogeneous populations, raising concerns about the generalizability of their findings to diverse populations.^16^ In addition to these, a robust association was discovered between a variant in the SORT1 gene and AAA, elucidating an intriguing link between lipid metabolism and AAA risk.^17^ Moreover, genome-wide association studies have unearthed risk loci on chromosomes 9p21 and 19q13, where genes with roles in the regulation of vascular smooth muscle cells reside.^18^ It's crucial to mention, though, that the precise mechanisms by which these loci influence AAA risk remain inadequately elucidated, indicating an area ripe for further exploration.

These diverse genetic influences highlight the multifactorial nature of AAA, involving a complex interplay of inflammatory pathways, extracellular matrix remodeling, lipid metabolism, and vascular cell regulation. In this labyrinth of genetic influences, the +276G/T and -420C>G polymorphisms have emerged as points of interest. While their role in AAA has not been comprehensively explored, their known involvement in inflammation and vascular remodeling ‒ processes central to AAA pathogenesis - makes them compelling candidates for investigation. Previous studies have hinted at these polymorphisms' relevance but lacked a comprehensive multi-factorial analysis that incorporates potential confounders and effect modifiers. The present study, therefore, offers new insights into the influence of these polymorphisms on AAA, adding to the nuanced understanding of the genetic landscape of this complex disease.

Hardy-Weinberg equilibrium, a cornerstone of population genetics, offers a means to determine whether the observed frequency of genotypes aligns with the expected distribution in a large, random-mating population. The compliance of both the +276G/T and -420C>G polymorphisms to this equilibrium in the present study confirms the representativeness of these samples. This validation establishes a solid ground for interpreting our results and their applicability to the wider population.

The lack of significant differences in the +276G/T genotype distribution between AAA patients and controls suggests that this polymorphism may not be a key player in AAA's genetic landscape. Previous studies have implicated the +276G/T polymorphism in other inflammatory conditions, highlighting its potential role in mediating inflammatory responses. For instance, it has been associated with increased risk and severity in conditions such as rheumatoid arthritis and systemic lupus erythematosus, diseases characterized by a significant inflammatory component.^19^ However, the role of the +276G/T polymorphism may not translate identically across different diseases. In the case of AAA, the pathogenesis involves not just inflammation but also vascular remodeling, extracellular matrix degradation, and potentially unique genetic regulation patterns. For instance, studies have shown that in atherosclerosis, another vascular inflammatory condition, the role of +276G/T polymorphism is less clear, with findings varying based on population and disease severity.^20^ The discrepancy between this finding and previous studies could stem from these differences in the genetic regulation and pathogenesis of these diseases, as well as the specific interactions between +276G/T and other genetic and environmental factors in AAA. This highlights the importance of disease-specific investigations in genetic research and underscores the complex nature of genetic influences in multifactorial diseases like AAA.

The -420C>G polymorphism demonstrated a significantly different distribution in AAA patients compared to controls, and the present regression analysis provided further insights into the effects of individual genotypes. Specifically, the CC genotype and CC/CG genotypes were associated with an increased risk of AAA, while the CC/CG genotypes were linked to an increased risk of AAA progression. However, the roles of the GG genotype and the combination of CC and GG genotypes were less clear, which underscores the complexity of the genetic factors involved in AAA. The potential role of the -420C>G polymorphism in AAA could be attributed to its location in the promoter region of the resistin gene. Variations in this region could influence gene expression, which might subsequently affect the production of resistin.^21^ Resistin has been implicated in several processes critical to AAA development, including inflammation and vascular dysfunction. This proposed mechanism finds support in studies conducted on other vascular diseases. For example, in diseases such as atherosclerosis and hypertension, polymorphisms in the promoter region of genes have been linked to altered gene expression and disease susceptibility.^22^, ^23^, ^24^ Specifically, the -420C>G polymorphism has been associated with resistin levels and vascular inflammation in these conditions. The present study results align well with these findings, suggesting a potential role of the -420C>G polymorphism in the development and progression of AAA. Nevertheless, the exact mechanisms underlying the observed associations remain to be clarified. The association between specific -420C>G genotypes and AAA underlines the importance of genotype-level analysis and emphasizes the need for further investigation into the role of this polymorphism in AAA.

## Conclusion

In the complex etiology of Abdominal Aortic Aneurysm (AAA), genetic factors are pivotal. Through a comprehensive retrospective analysis comparing AAA patients to healthy controls, this research emphasized the potential influence of the resistin gene's +276G/T and -420C>G polymorphisms. While the +276G/T polymorphism showed no pronounced variance between the two cohorts, the genotype distribution of the -420C>G polymorphism demonstrated notable disparities. Notably, individuals with the CC genotype and those with CC/CG genotypes of the -420C>G polymorphism appear more susceptible to AAA. Thus, the present research suggests a significant contribution of the -420C>G polymorphism, especially the CC and CC/CG genotypes, in the onset of AAA. Further studies are paramount to solidify these connections and delve deeper into the resistin gene's implications in AAA pathogenesis.

## Conflicts of interest

The authors declare no conflicts of interest.
